# MammaPrint guides treatment decisions in breast Cancer: results of the IMPACt trial

**DOI:** 10.1186/s12885-020-6534-z

**Published:** 2020-01-31

**Authors:** Hatem Soliman, Varsha Shah, Gordan Srkalovic, Reshma Mahtani, Ellis Levine, Blanche Mavromatis, Jayanthi Srinivasiah, Mohamad Kassar, Robert Gabordi, Rubina Qamar, Sarah Untch, Heather M. Kling, Tina Treece, William Audeh

**Affiliations:** 1 Moffitt Cancer Center, Tampa, FL USA; 2 Ascension Columbia St. Mary’s Hospital, Milwaukee, WI USA; 3 Herbert-Herman Cancer Center, Sparrow Hospital, Lansing, MI USA; 4 University of Miami, Miami, FL USA; 5 Roswell Park Cancer Institute, Buffalo, NY USA; 6 Western Maryland Health System, Cumberland, MD USA; 7 Emory Decatur Hospital, Decatur, GA Georgia; 8 Community Hospital Northwest Oncology, Munster, IN USA; 9 St. Joseph’s Women’s Hospital, Tampa, FL USA; 10 Aurora Health Care, Milwaukee, WI USA; 11 Agendia, Irvine, CA USA

**Keywords:** Breast cancer, Diagnostic test, MammaPrint, BluePrint, 70-GS, 80-GS, Molecular profiling, Clinical utility

## Abstract

**Background:**

Increased usage of genomic risk assessment assays suggests increased reliance on data provided by these assays to guide therapy decisions. The current study aimed to assess the change in treatment decision and physician confidence based on the 70-gene risk of recurrence signature (70-GS, MammaPrint) and the 80-gene molecular subtype signature (80-GS, BluePrint) in early stage breast cancer patients.

**Methods:**

IMPACt, a prospective, case-only study, enrolled 452 patients between November 2015 and August 2017. The primary objective population included 358 patients with stage I-II, hormone receptor-positive, HER2-negative breast cancer. The recommended treatment plan and physician confidence were captured before and after receiving results for 70-GS and 80-GS. Treatment was started after obtaining results. The distribution of 70-GS High Risk (HR) and Low Risk (LR) patients was evaluated, in addition to the distribution of 80-GS compared to IHC status.

**Results:**

The 70-GS classified 62.5% (*n* = 224/358) of patients as LR and 37.5% (*n* = 134/358) as HR. Treatment decisions were changed for 24.0% (*n* = 86/358) of patients after receiving 70-GS and 80-GS results. Of the LR patients initially prescribed CT, 71.0% (44/62) had CT removed from their treatment recommendation. Of the HR patients not initially prescribed CT, 65.1% (41/63) had CT added. After receiving 70-GS results, CT was included in 83.6% (*n* = 112/134) of 70-GS HR patient treatment plans, and 91.5% (*n* = 205/224) of 70-GS LR patient treatment plans did not include CT. For patients who disagreed with the treatment recommended by their physicians, most (94.1%, *n* = 16/17) elected not to receive CT when it was recommended. For patients whose physician-recommended treatment plan was discordant with 70-GS results, discordance was significantly associated with age and lymph node status.

**Conclusions:**

The IMPACt trial showed that treatment plans were 88.5% (*n* = 317/358) in agreement with 70-GS results, indicating that physicians make treatment decisions in clinical practice based on the 70-GS result. In clinically high risk, 70-GS Low Risk patients, there was a 60.0% reduction in treatment recommendations that include CT. Additionally, physicians reported having greater confidence in treatment decisions for their patients in 72% (*n* = 258/358) of cases after receiving 70-GS results.

**Trial registration:**

“Measuring the Impact of MammaPrint on Adjuvant and Neoadjuvant Treatment in Breast Cancer Patients: A Prospective Registry” (NCT02670577) retrospectively registered on Jan 27, 2016.

## Introduction

The inclusion of chemotherapy in treatment recommendations for early stage breast cancer patients is largely based on the estimated risk of distant recurrence or metastasis. The accurate identification of patients with a high risk of recurrence would thus indicate that some form of targeted or systemic chemotherapy is of clinical value. Likewise, avoiding overtreatment of patients who would not benefit from inclusion of chemotherapy in their treatment regimen is paramount. Risk of recurrence in early stage breast cancer varies widely from patient to patient, and estimation of risk has historically relied on a combination of clinical and pathologic factors, such as tumor grade, size, stage, lymph node involvement, estrogen receptor (ER), progesterone receptor (PR), human epidermal growth factor receptor 2 (HER2) status, and patient (age, menopausal status) characteristics. However, the use of genomic profiling assays to estimate risk of recurrence has increased in recent years, and these types of assays may provide a more precise prognosis based on the biology of the tumor [1].

The 70-gene signature (70-GS, MammaPrint) is a microarray-based, FDA-cleared, molecular diagnostic assay that assigns tumors into categories of high or low risk of metastasis based on the combined expression of 70 genes [2–5]. The 70-GS was developed independently of clinical pathology by interrogating ~ 25,000 genes representing the entire human genome for a gene expression signature associated with disease outcome [5], and validated in the prospective, randomized ‘Microarray in Node-negative and 1 to 3 positive lymph node Disease may Avoid Chemotherapy’ (MINDACT) trial [6]. The 80-gene signature (80-GS, BluePrint) is a molecular subtyping microarray-based assay that uses the combined expression of 80 genes to categorize tumors as Luminal-, HER2- or Basal-type [7–9], and when combined with the 70-GS, categorizes Luminal-type tumors as Luminal A (Low Risk) or B (High Risk).

Utility of the 70-GS in clinically high risk patients has been demonstrated by the MINDACT trial, in which 46% of patients at high risk of distant recurrence using clinical factors were classified as genomically Low Risk by the 70-GS [6]. These patients did not significantly benefit from chemotherapy in the randomized arm of the trial. Additionally, in the Neoadjuvant Breast Registry Symphony Trial (NBRST), Luminal A patients had low rates of pathological complete response but comparatively good clinical outcomes, which supports that MP Low Risk patients do not benefit from neoadjuvant chemotherapy [8].

Increased usage of genomic assays suggests an increased reliance on the data provided, and impact studies may be informative in evaluating the extent of physician adherence to assay results in guiding therapy decisions. A previous prospective study, PROMIS, determined that the 70-GS led to a change in treatment recommendations in 33.6% of patients who had an intermediate risk result by the 21-gene assay (21-GA, Oncotype DX) and an increase in physician confidence [10]. Here, the prospective IMPACt trial aimed to measure the effect of 70-GS and 80-GS results on physicians’ chemotherapy treatment decisions for all early-stage, hormone receptor-positive, human epidermal receptor-negative (HER2-) patients, not only intermediate 21-GA patients. IMPACt also evaluated patient characteristics associated with treatment recommendations that were discordant with 70-GS results, as well as patient treatment decisions in comparison with physician recommendations. Here, we report the results of this trial, including overall change in treatment recommendation and physician confidence in treatment plans.

## Patients and methods

### Study population

The prospective study for Measuring the Impact of MammaPrint on Adjuvant and Neoadjuvant Treatment in Breast Cancer Patients: A Prospective Registry (IMPACt) was approved by institutional review boards at all 18 participating US institutions (NCT02670577). This observational study enrolled 452 breast cancer patients between November 2015 and August 2017. The primary objective population included patients with histologically-proven stage I or II, hormone receptor-positive (defined as ER-positive or PR-positive or both, according to local assessment) and HER2-negative (immunohistochemistry [IHC] 0–1+ or FISH/ISH non-amplified according to local assessment) breast cancer, with 0–3 involved axillary lymph nodes (pN0/N1, macro-metastases > 2 mm or micro-metastases 0.2-2 mm) with target enrollment of 331 patients receiving adjuvant therapy. ER and PR were considered positive if ≥1% of tumor cells demonstrated positive nuclear staining by IHC, as determined by institutional pathology laboratory assessment. The primary objective of the study was to assess the impact of 70-GS/MammaPrint results on adjuvant treatment decisions for these patients. To be eligible for enrollment, patients should have been eligible to receive chemotherapy and endocrine therapy as defined by a good Karnofsky index (≥ 80) and be free of hematologic, cardiologic, or hepatic contraindications, or any impeding comorbidity. Prior to enrollment, patients provided written informed consent to participate in the registry, for research use of their tumor samples, and for collection of clinical data. Patients were required to be ≥18 years of age at the time of consent.

Patients with triple-negative and HER2-positive (regardless of hormone receptor status) breast cancer could be included to address secondary objectives, which included assessment of the impact of 70-GS and 80-GS on treatment decisions in T1a/b N0/N1 triple negative and HER2-positive patients, the impact of 70-GS and 80-GS on neoadjuvant treatment decisions, and to compare clinical subtype based on IHC/FISH estrogen receptor (ER), progesterone receptor (PR), HER2, and Ki-67 with 80-GS molecular subtype. To be included in this study arm, patients were required to have histologically proven invasive T1a or T1b breast cancer, which was hormone receptor negative (ER and PR) by local standards and negative (IHC 0–1+ or FISH/ISH non-amplified) or positive (IHC 3+ or FISH/ISH amplified) by local assessments, with 0–1 involved axillary lymph nodes (macro-metastases > 2 mm or micro-metastases 0.2-2 mm). Target enrollment for assessing the secondary objective of the impact of 70-GS and 80-GS on chemotherapy decisions was 50 triple-negative breast cancer patients, 50 HER2-positive breast cancer patients, and 50 patients receiving neoadjuvant therapy. The analyses for this secondary objective would only be performed if the target enrollment was achieved.

Patients were excluded from study participation if they had a previous diagnosis of a breast malignancy, unless disease free for at least 10 years, metastatic disease, a tumor sample that failed QA/QC criteria for 70-GS/80-GS testing, or had started or completed adjuvant or neoadjuvant chemotherapy for current breast cancer.

### Molecular risk profile assessment and molecular subtyping

The 70-GS and 80-GS tests, which evaluate breast tumor tissue RNA expression using custom microarray chips (Agilent Technologies, city, CA, USA), were performed according to standard protocols as previously described [9, 11] at the centralized Agendia laboratory (Irvine, CA). To obtain a valid result, a 30% minimum tumor composition of the tissue sample was required. The 70-GS test results provide an index score between − 1.000 and 1.000 and categorize tumors as Low (index of 0.001 to 1.000) or High (index − 1.000 to 0) Risk of recurrence. The correlation of a patient’s tumor gene expression profile to known Low and High Risk profiles is used to calculate the 70-GS index value. When used in combination with 70-GS, the molecular subtyping assay 80-GS classifies tumors into the following subtypes: Luminal A, Luminal B, HER2-type, and Basal-type.

### Clinical risk assessment

Clinical risk was determined using a combination of clinical pathologic factors including tumor size, lymph node involvement, histologic grade, ER, and HER2 status [6]. Based on this assessment, tumors were classified as either low or high risk of recurrence. Clinically low risk tumors included those that were ER-positive, HER2-negative, negative for lymph node involvement, and up to 3 cm if well-differentiated (grade 1), up to 2 cm if moderately differentiated (grade 2), or up to 1 cm if poorly differentiated (grade 3). Tumors were also considered to be clinically low risk with up to 3 positive lymph nodes if grade 1 and no more than 2 cm in size. Tumors of any size were considered clinically high risk if positive for nodal involvement and either ER-negative or HER2-positive. However, HER2-positive tumors were also considered to be clinically low risk if negative for nodal involvement, grade 1 or 2, and up to 2 cm if ER-positive, or up to 1 cm if ER-negative. ER-negative, HER2-negative tumors were considered clinically high risk if positive for nodal involvement; however, if node-negative, considered clinically low risk if were grade 1, up to 2 cm, or grade 2, up to 1 cm.

### Physician confidence assessment

Physician confidence in treatment plan was recorded on a paper questionnaire, and then reported on a standard case report form (CRF). Physicians were asked to rate confidence level on a scale of − 2 to + 2, where 0 is neutral. The questionnaires measuring confidence were not validated; they represent the subjective opinion of the physicians.

### Statistical analysis

The IMPACt study was powered to detect a 25% overall treatment change (5% two-sided significance and 95% power) in patients receiving adjuvant chemotherapy or endocrine therapy. At a 5% significance level, the sample size required to investigate the hypothesis was calculated to be 301 stage I and II hormone receptor-positive and HER2-negative breast cancer patients. Taking into account an estimated drop-out rate of 10%, the estimated required sample size was 331 patients. The IMPACt study enrolled 358 stage I-II, hormone receptor-positive, HER2-negative breast cancer patients, and was thus sufficiently powered to investigate the hypothesis of a 25% treatment change after disclosure of 70-GS results to the investigator.

The overall change in treatment recommendation is expressed as a percentage of primary objective patients (stage I-II, hormone receptor-positive, HER2-negative, *n* = 358) whose post 70-GS treatment recommendations were changed from their pre 70-GS treatment recommendation. To compare patient and tumor characteristics between 70-GS Low Risk and High Risk groups, χ^2^ test (for binary variables, > 2 groups) or unpaired two-tailed Student *t* test (for age as a continuous variable, 2 groups) were used. To determine clinical pathologic factors that were associated with discordant treatment recommendations (i.e., when CT was recommended for genomically Low Risk patients or not recommended in High Risk patients), multivariate logistic regression analysis was performed. A *p*-value of *p* < 0.05 was considered significant. Statistical tests were performed with Prism version 7.02 (Graphpad, La Jolla, CA, US) or with SPSS 22.0 for Windows (SPSS Inc., Chicago, IL, US).

## Results

### Patient and tumor characteristics

**Fig. 1:**
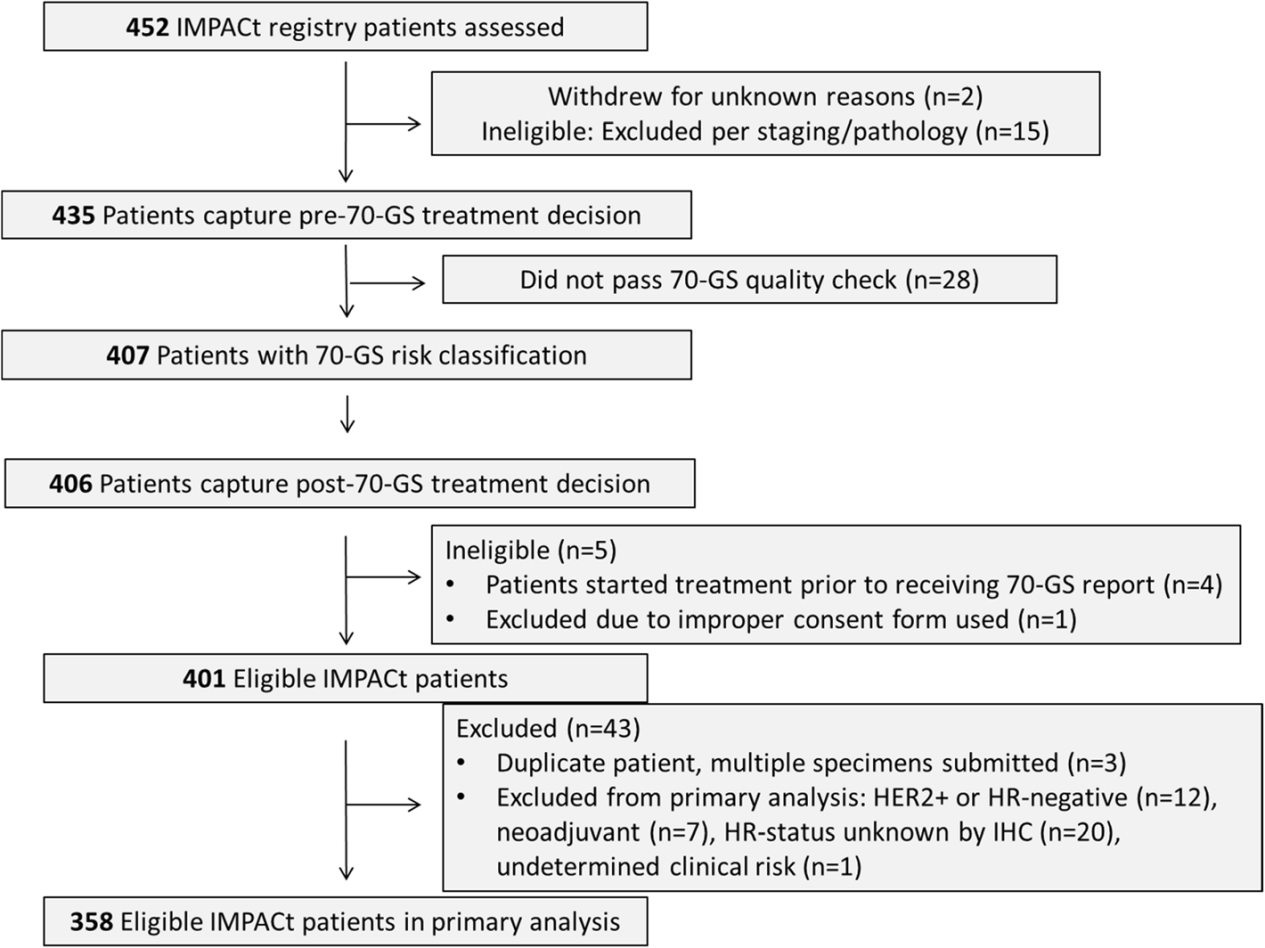
Consort diagram of IMPACt study. Numbers of patients excluded from the primary objective study population and reasons for exclusion are indicated

From November 2015 to August 2017, 452 patients were enrolled in the prospective IMPACt registry. Patients excluded from the primary analysis (Consort diagram, Fig. 1) included tumor specimens that did not pass 70-GS quality check (*n* = 28), screening failures (*n* = 38), those who began treatment prior to receiving the 70-GS report (*n* = 4), those with unknown hormone receptor status by IHC, unknown treatment decision, or insufficient information to determine clinical risk (*n* = 22), and those who withdrew from the study (*n* = 2). This resulted in 358 eligible patients in the primary objective population of hormone receptor-positive, HER2-negative, stage I-II patients who planned to receive systemic adjuvant therapy. The primary study population was 75.7% (*n* = 271/358) post-menopausal and 80.2% (*n* = 287/358) Caucasian, with a mean age of 60.9 and a median of 62 (range = 30–84) years (Table 1). The majority of the tumors were T1 (*n* = 277/358, 77.4%) and moderately differentiated (grade 2, *n* = 192/358, 53.6%). Lymph node involvement was reported in 80/358 patients (22.3%).

**Table 1:** Patient Characteristics by 70-GS Result

Patient Characteristics	70-GS Result			
Age (yrs)	Low Risk (*n* = 224)	High Risk (*n* = 134)	Total (*n* = 358)	*p*- value
median	62	62		*p* = 0.388
mean	61.4	60.0		
Clinical Risk				
Low Risk	160 (70.5%)	67 (20.5%)	227	*p* < 0.0001
High Risk	64 (48.9%)	67 (51.1%)	131	
Menopausal Status				
Post	173 (63.8%)	98 (36.2%)	271	*p* = 0.632
Pre/Peri	45 (60.8%)	29 (39.2%)	74	
Unknown	6 (46.2%)	7 (53.8%)	13	
Tumor Stage				
T1 (all)	179 (64.6%)	98 (35.4%)	277	*p* = 0.585
*T1a*	*15 (62.5%)*	*9 (37.5%)*	*24*	
*T1b*	*59 (65.6%)*	*31 (34.4%)*	*90*	
*T1c*	*96 (63.6%)*	*55 (36.4%)*	*151*	
T2	44 (55.0%)	36 (45.0%)	80	
T3	1 (100%)	0 (0%)	1	
Nodal Status				
N0	173 (62.2%)	105 (37.8%)	278	*p* = 0.896
N1	51 (63.8%)	29 (36.3%)	80	
Grade				
G1: Low grade	85 (85.0%)	15 (15.0%)	100	*p* < 0.0001
G2: Intermediate grade	122 (63.5%)	70 (36.5%)	192	
G3: High grade	14 (22.2%)	49 (77.8%)	63	
GX: Unknown	3 (100%)	0 (0%)	3	
Tumor Type				
Invasive ductal carcinoma (IDC)	161 (59.4%)	110 (40.6%)	271	*p* = 0.032
Invasive lobular carcinoma (ILC)	41 (80.4%)	10 (19.6%)	51	
IDC/ILC	5 (5.0%)	5 (50.0%)	10	
Other	17 (65.4%)	9 (34.6%)	26	
Ethnicity				
African/Black	23 (67.6%)	11 (32.4)	34	*p* = 0.603
Caucasian/White	181 (63.1%)	106 (36.9%)	287	
Hispanic	10 (50.0%)	10 (50.0%)	20	
Other	10 (58.8%)	7 (41.2%)	17	
PR Status (IHC)				
IHC Negative	11 (32.4%)	23 (67.6%)	34	*p* = 0.0003
IHC Positive	212 (65.5%)	111 (34.5%)	323	
Unknown	1 (100%)	0 (0%)	1	

Patients whose tumors were HER2-positive (*n* = 8) or triple-negative (*n* = 4) by histology, patients who could not be staged (*n* = 6), those being treated in the neoadjuvant setting (*n* = 7) or those with an unknown treatment decision (*n* = 1) were not included in the primary analysis, but were included in the molecular subtyping analysis (Additional file 1: Table S1, *n* = 384, combined with primary Stage I-II, hormone receptor-positive, HER2-negative population). The study did not enroll sufficient numbers of triple-negative (*n* = 4), HER2-positive (*n* = 8), and neoadjuvant patients (*n* = 7) to assess the impact of 70-GS and 80-GS on chemotherapy decisions in these populations, per the secondary objective of the study. However, molecular subtype classification by 80-GS was compared with conventional subtype assessment (Additional file 1: Table S1), and the total frequency of subtype reclassifications was 39.3% (*n* = 152/384).

### Clinical risk assessment and physician treatment plan prior to 70-GS results in the primary objective population, stage I-II, hormone receptor-positive, HER2-negative patients

**Fig. 2:**
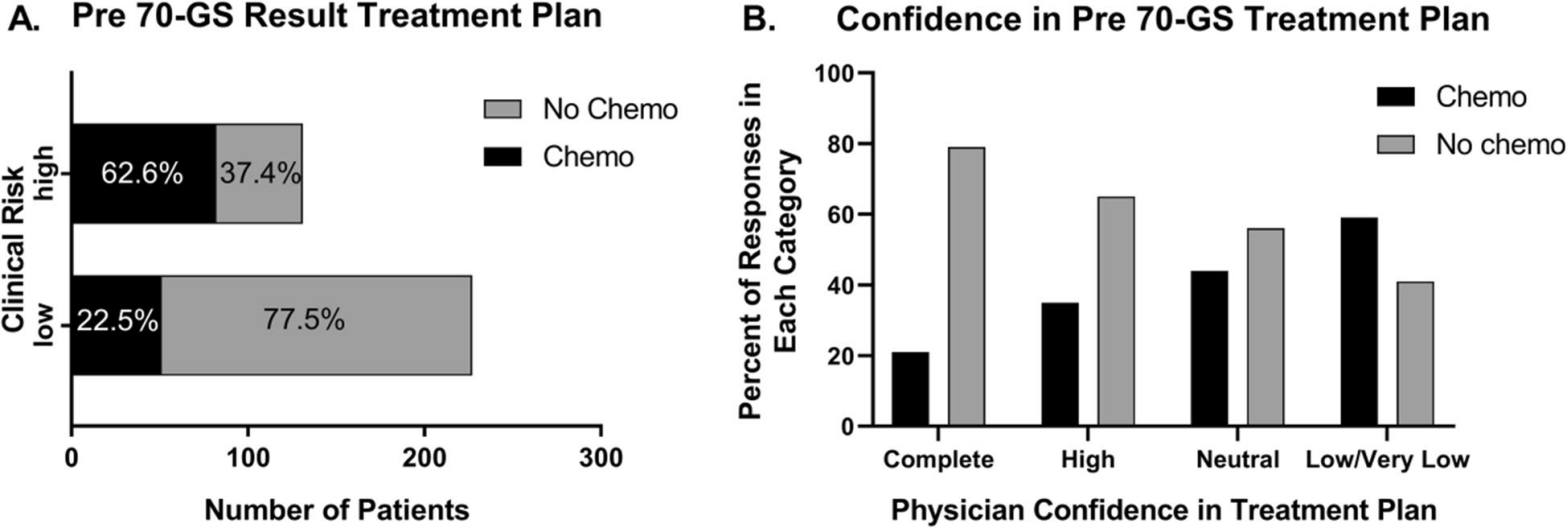
Treatment Plan and Physician Confidence Prior to 70-GS Results. Numbers and percentages of clinically low risk and high risk patients whose physicians planned, prior to receiving 70-GS results, to include or not include chemotherapy as part of their treatment plans are shown (**a**). There were 176/ 227 clinically low risk patients whose physicians did not include chemotherapy in their treatment plans and 82/131 clinically high risk patients whose physicians included chemotherapy in their treatment plans. Physician confidence in treatment plans, prior to 70-GS result, is shown as a percentage of plans that include chemotherapy or not in each confidence category (complete, high, neutral, and low/very low, *p* < 0.0001, (**b**))

According to clinical risk assessment using the MINDACT criteria [6], 63.4% (*n* = 227/358) of patients were classified as low risk, and 36.6% (*n* = 131/358) of patients were classified as high risk of distant recurrence (Table 1, Fig. 2a). For clinically low risk patients, 77.5% (176/227) were recommended not to receive chemotherapy by their physicians; whereas 62.6% (82/131) of clinically high risk patients were recommended treatment plans that included chemotherapy (Fig. 2a). Physician-reported confidence in treatment plans is given in Fig. 2b; and greater confidence was associated with treatment plans that did not include chemotherapy (*p* < 0.0001).

### 70-GS risk classification and impact on treatment decisions for stage I-II, hormone receptor-positive, HER2-negative patients

**Fig. 3:**
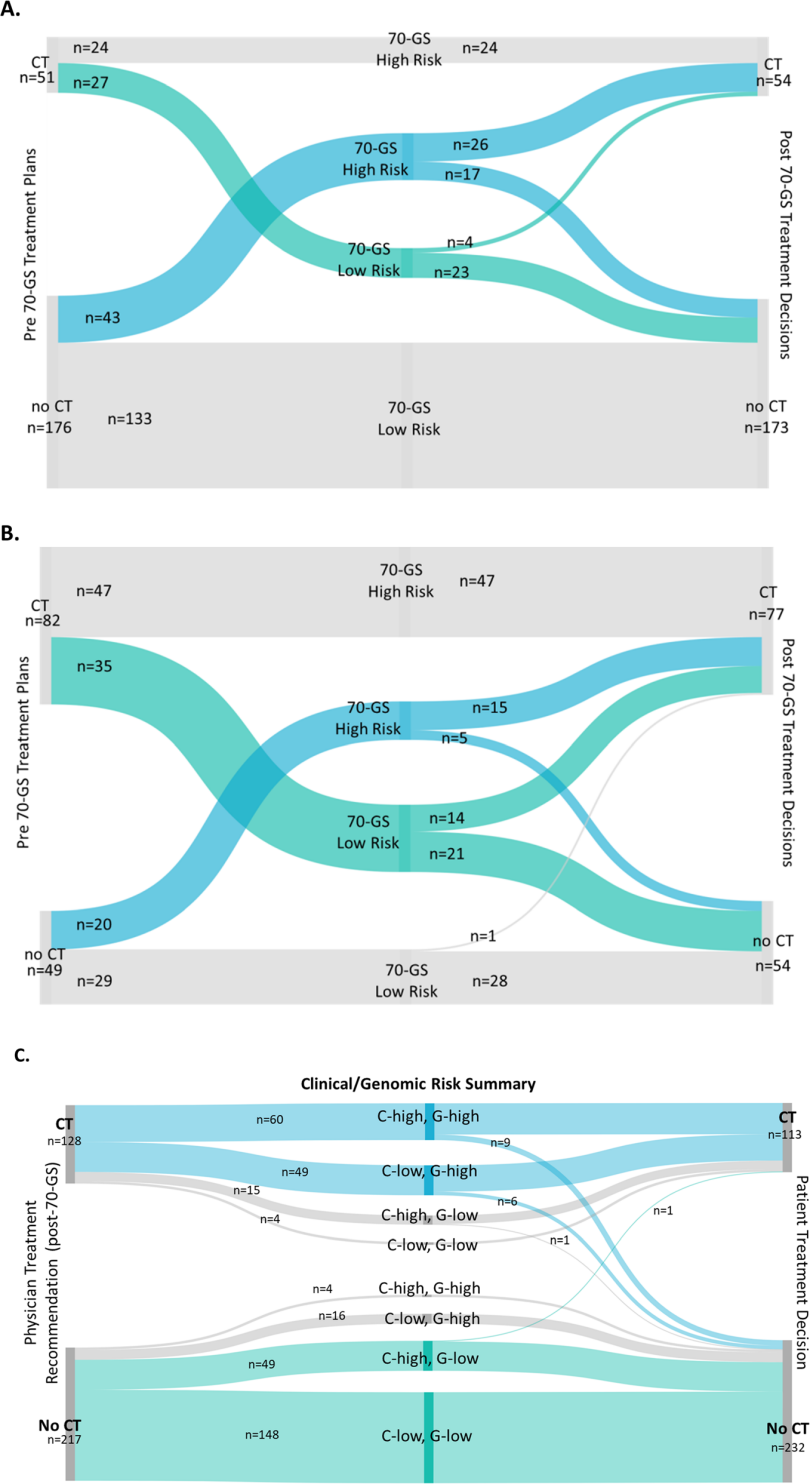
Change in treatment recommendations from pre-70-GS to post-70-GS results and patient agreement with physician recommendations. Numbers of patients in each category (70-GS High Risk, 70-GS Low Risk, pre-70-GS treatment recommendation, and post-70-GS treatment recommendation) are shown for clinically low risk (**a**, *n* = 227) and clinically high risk (**b**, *n* = 131) patients. Treatment recommendations are indicated as inclusion or exclusion of chemotherapy (CT). Patient agreement/disagreement with physician-recommended treatment plan is shown in (**c**). Numbers of patients in each category are indicated, as well as the clinical/genomic risk stratification of the patients in each subset

The 70-GS classified 62.5% (*n* = 224/358) of patients as Low Risk and 37.5% (*n* = 134/358) as High Risk. After receiving 70-GS results, physicians elected to change the chemotherapy (CT) treatment recommendation in 24.0% (*n* = 86/358) of total cases. Post-70-GS treatment plans were 88.5% (*n* = 317/358) in agreement with 70-GS results [83.6% (*n* = 112/134) for CT in 70-GS High Risk patients; 91.5% (*n* = 205/224) for no CT in 70-GS Low Risk patients]. A summary of pre- and post-70-GS treatment recommendations in clinically low risk (Fig. 3a) and clinically high risk (Fig. 3b) patients shows the numbers of patients in each category for whom recommendations were or were not changed following the 70-GS result. In the group of patients with clinically high risk, 70-GS Low Risk (Table 2, “C-high, G-low”) tumors, which was the primary test population in the MINDACT trial [6], physicians removed CT from treatment recommendations for 60.0% (21/35) of patients for whom it was initially recommended (Fig. 3b).

**Table 2:** Treatment plan recommendations pre- and post-70-GS results, according to clinical and genomic risk result category

	Treatment Decisions Pre- to Post-70-GS					
Clinical, Genomic Risk Summary	Chemo to Chemo	Chemo to No Chemo	No Chemo to Chemo	No Chemo to no Chemo	Total	*p*-value
C-low, G-low	4 (2.5%)	23 (14.4%)	0 (0.0%)	133 (83.1%)	160	*p* < 0.0001
C-high, G-low	14 (21.9%)	21 (32.8%)	1(1.6%)	28 (43.8%)	64	
C-low, G-high	24 (35.8%)	0 (0.0%)	26 (40.6%)	17 (26.6%)	67	
C-high, G-high	47 (70.1%)	0 (0.0%)	15 (23.4%)	5 (7.8%)	67	
Total	89 (24.9%)	44 (12.3%)	42 (11.7%)	183 (51.1%)	358	

Patient enrollment for IMPACt began prior to the publication of the MINDACT trial results [6] and continued up to 1 year following the publication; however, most (69.8%, *n* = 250/358) patients were enrolled after MINDACT results were published. Rates of concordance between physician treatment recommendation and 70-GS results were compared in patients enrolled before and after the MINDACT publication (Table 3). Although there was a trend toward increased concordance with 70-GS results in the group that was enrolled post-MINDACT, there was no significant difference in treatment recommendations. Concordance was generally higher in the 70-GS Low Risk group, which showed 92.6% (*n* = 150/162) concordance between treatment recommendation and 70-GS results in patients enrolled post-MINDACT publication (Table 3).

**Table 3:** Comparison of post-70-GS treatment recommendations prior to and following publication of MINDACT results, proportions of recommendations concordant or discordant with 70-GS results

Timing of Treatment Recommendations	Concordance of Treatment Recommendations with 70-GS Results			
All Patients	Concordant	Discordant	Total	*p*-value
Pre-MINDACT	91 (84.3%)	17 (15.7%)	108	*p* = 0.094
Post-MINDACT	226 (90.4%)	24 (9.6%)	250	
70-GS Low Risk Patients				
Pre-MINDACT	55 (88.7%)	7 (11.3%)	62	*p* = 0.351
Post-MINDACT	150 (92.6%)	12 (7.4%)	162	
70-GS High Risk Patients				
Pre-MINDACT	36 (78.3%)	10 (21.7%)	46	*p* = 0.229
Post-MINDACT	76 (86.3%)	12 (13.6%)	88	

Patient agreement with physician treatment plan was also assessed in the primary objective population (*n* = 345, unknown decisions excluded). Physicians were asked whether patients agreed with their treatment recommendation, and if not, whether the patient preferred to include chemotherapy or not. In the overall group assessment, patient treatment decisions were concordant with physician recommendations in 95.1% (*n* = 328/345) of cases (Fig. 3c). Most (88.2%, *n* = 15/17) of the discordant cases were 70-GS High Risk and nearly all (16/17) of these patients elected not to receive CT although it was recommended by their physician (Fig. 3c). In the 70-GS High Risk group not planning to receive CT prior to receiving the 70-GS result (*n* = 63), physicians modified their treatment plan to include CT in 65.1% (*n* = 41) of cases (Fig. 3a-b); however, 17.1% (*n* = 7/41) of these patients did not elect to add CT to their treatment plans (Fig. 3c). In the 70-GS Low Risk group planning to receive CT prior to receiving the test result (*n* = 62), physicians modified the treatment plan to remove CT in 71.0% (*n* = 44/62) of cases (Table 2, Fig. 3a-b), and all except for one of the patients agreed with the physician recommendation. Additionally, of the 70-GS Low Risk patients previously planning to receive chemotherapy, one patient decided to remove chemotherapy despite her physician’s recommendation to include it (Fig. 3c).

### 70-GS risk classification result impact on physician confidence in stage I-II, hormone receptor-positive, HER2-negative patients

**Fig. 4:**
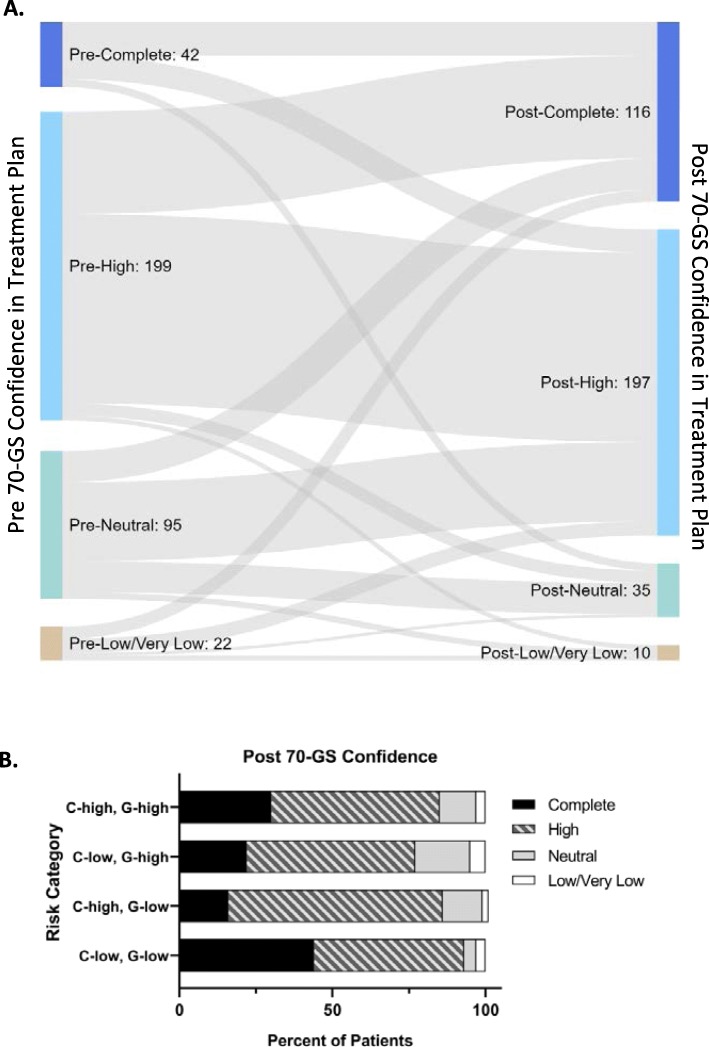
Physician Change in Confidence in Treatment plans post 70-GS Result. Change in physician confidence in treatment plan shown in a Sankey diagram, with physician-reported confidence (complete, high, neutral, or low/very low) in patient treatment plans prior to receiving 70-GS result on the origins on the left side of the diagram, and the changes in those responses following 70-GS results on the endpoints, shown on the right side of the diagram (**a**). Numbers in each confidence category prior to 70-GS (left side) and post-70-GS (right side) are given. The width of each flow line is proportional to the number of responses in that category. In **b**, Post 70-GS confidence categories are shown as a percent of each combined risk category (clinical risk, determined by MINDACT criteria, combined with genomic risk, determined by 70-GS result). Numbers of patients in each category are as follows: 160 C-low/G-low, 64 C-high/G-low, 67 C-low/G-high, 67 C-high/G-high

Physicians were queried regarding their confidence in patient treatment plan prior to and following the 70-GS result, and these responses are summarized in Fig. 4a. There were 52 physicians who enrolled patients in the primary objective population, all of whom provided responses regarding confidence in treatment plan. The number of physician responses of complete confidence in treatment plan increased by 2.8-fold from pre-70-GS (*n* = 42/358) to post-70-GS (*n* = 116/358) result (Fig. 4a). Additionally, the number of physician responses of low or very low confidence in treatment plan decreased by 54.5% from pre-70-GS result (*n* = 22/358) to post-70-GS (*n* = 10/358) result. After receiving the 70-GS result, physicians were queried regarding the impact of the 70-GS result on their confidence in the treatment plan selected. They selected from one of the following responses: slightly or significantly increasing confidence, slightly or significantly lessening confidence or having no effect. Physicians reported an increase (either significant or slight) in confidence in treatment decision in 72.1% (*n* = 258/358) of cases. Percent of each confidence category (complete, high, neutral, low/very low) was evaluated by combined clinical and genomic risk categories (Fig. 4b, *n* = 358 total responses). Physician-reported post-70-GS confidence in treatment plan was greatest in concordant low risk (clinical low risk, 70-GS Low Risk, *n* = 149/160 responses in “complete” or “high” categories) patients (Fig. 4).

### Patient characteristics associated with discordant treatment plans in stage I-II, hormone receptor-positive, HER2-negative patients

Physicians’ treatment plans disagreed with the 70-GS result in 11.5% of patients overall, e.g., 70-GS Low Risk patients whose physicians recommended including CT in treatment plans. Patient and tumor characteristics were evaluated in these patients, and results from multivariate logistic regression analysis are shown in Table 4. Age, grade, and lymph node status were significantly associated with cases of physician-recommended treatment plans that were discordant with the 70-GS result. In the 70-GS Low Risk group, patients for whom physicians recommended chemotherapy tended to be younger at diagnosis, have a positive lymph node status, and/or have a tumor with higher histopathologic grade. In the 70-GS High Risk group, patients for whom physicians did not recommend chemotherapy were older and/or had no lymph node involvement. Patients with grade 3 tumors were more likely to be recommended chemotherapy; thus, there was a greater concordance between 70-GS result and chemotherapy treatment for patients with grade 3 tumors compared with those with lower grade tumors.

**Table 4:** Multivariate logistic regression models for the discordant treatment plans in 70-GS Low Risk and High Risk patients

Variable				95% CI	
70-GS Low Risk Patients (*n* = 215)		*p*-value	OR	Lower	Upper
Age		0.105	0.934	0.861	1.014
Menopausal Status	Post vs. pre/peri	0.650	0.672	0.120	3.750
Tumor Stage	2 vs. 1	0.155	2.504	0.706	8.880
Nodal Status	1 vs. 0	0.003	7.957	1.884	22.512
Grade	2/3 vs. 1	0.051	5.106	0.993	26.245
70-GS High Risk Patients (*n* = 135)		*p*-value	OR	Lower	Upper
Age		0.033	1.050	1.004	1.099
Nodal Status	1 vs. 0	0.048	0.121	0.015	0.978
Grade	2/3 vs. 1	0.086	0.335	0.096	1.166

## Discussion

The IMPACt trial sought to measure the effects of 70-GS/80-GS (MammaPrint/BluePrint) testing on physicians’ treatment recommendations, in particular, the use of adjuvant chemotherapy and their confidence in those recommendations. Change in treatment plan was significantly associated with 70-GS result, and after receiving the 70-GS results, treatment plans were 88.5% in agreement with test results (83.6% in 70-GS High Risk patients; 91.5% in 70-GS Low Risk patients). When the physician-recommended plans did not agree with 70-GS results, factors that were significantly associated with discordance included the patient’s age, lymph node involvement, and histopathologic grade. Overall, the study demonstrates that physicians changed their treatment recommendation for systemic chemotherapy in 24.0% of cases after receiving the 70-GS result. In 70-GS Low Risk patients, physicians removed CT from the treatment recommendation for the majority (60.0%) of patients for whom they had previously recommended CT.

The first study to prospectively evaluate the performance of the 70-GS in a community setting was the microarRAy prognoSTics in breast cancER (RASTER) study [12, 13], which was also the first study to assess the impact of a gene expression prognosis classifier on adjuvant therapy decision making. In the RASTER study, similar to the current IMPACt study, patients were stratified by clinical risk and 70-GS risk assessment. The clinical risk assessment was performed using Adjuvant! software, which was also the basis for the MINDACT clinical risk assessment [6]. The 70-GS classified 42.0% (124/295) of clinically high risk were classified as Low Risk by the 70-GS, and 76% of these patients did not receive adjuvant chemotherapy, notably with excellent outcomes (98% survival without recurrence at 5 years) [12]. This is similar to IMPACt, in which 76.6% (49/64) of clinically high risk, 70-GS Low Risk patients elected to omit chemotherapy from their treatment plans. Other trials have examined impact of the 70-GS test in European countries [14, 15]. One of these, PRospective Study to Measure the Impact of MammaPrint on Adjuvant Treatment in Hormone Receptor-positive HER2-negative Breast Cancer Patients (PRIMe), conducted primarily in Germany, reported a 29.1% rate of change in treatment recommendation and rates of physician adherence to 70-GS risk assessment of 92.3% in 70-GS Low Risk and 94.3% in 70-GS High Risk cases [14, 16], similar to reported rates in the current study. The patient population in PRIMe was similar to IMPACt, mostly post-menopausal patients with tumors that were most typically T1 and predominantly negative for lymph node involvement [14]. This study reported high rates of adherence in discordant groups, with 74.7% of physicians removing CT from treatment plans that initially included it for patient with Low Risk 70-GS results and 88.9% of physicians who initially omitted CT and changed their recommendation to include CT following a 70-GS High Risk result [14]. In another prospective impact study, The Symphony Triple A Study: Using Symphony in Treatment Decisions Concerning Adjuvant Systemic Therapy, conducted in the Netherlands, the authors report that treatment recommendations were changed for 51.5% of patients after receiving the 70-GS result, and the CT recommendation agreed with the 70-GS result in 96% of cases [15]. Although this rate of change in CT recommendation appears higher than in the current study or in PRIMe, the difference in study design should be noted, as 42.9% of physicians did not provide a treatment recommendation prior to 70-GS result in this study [15]. This rate of change in treatment recommendation only includes the physicians who provided a recommendation prior to receiving 70-GS results.

Results from the current study suggest that patients were less likely to agree to a treatment plan that includes CT, despite the potential clinical benefit. In most cases (95.1%), patient decisions were concordant with physician recommendations. However, most discordant decisions were in favor of excluding CT from treatment plans, despite a High Risk result by 70-GS. In 70-GS High Risk cases in which CT was not included in the original treatment plan, but added by physicians following the 70-GS result, 17.1% of these patients did not agree to include CT in their treatment. In the current study, we did not capture the explanation for a patient’s decision to exclude CT; however, we can speculate that High Risk patients who elected not to receive CT may have considered other factors aside from the potential clinical benefit of this treatment, such as immediate side effects and adverse events, long-term effects and co-morbidities, and the impact on their quality of life [17]. Patients may be concerned about adverse clinical events, including both short-term effects, such as nausea, vomiting, fever, infections, myelosuppression, hair loss, cytopenia, etc., and long-term impacts on quality of life, such as cognitive limitations, fatigue, pain, neuropathy, depression/anxiety, cardiac function/dysfunction, premature menopause, and sexual dysfunction [18–23]. Patients may also be concerned about potential financial toxicity of CT. Cost of cancer care in the United States has increased two to three times faster than other health care costs in recent years, and is projected to incur an annual cost of $173 billion by 2020, nearly a 40% increase over 2010 annual costs [24]. Increases in patients’ out-of-pocket expenses have been associated with serious financial hardship and distress, including bankruptcy [25, 26], leading to reduced adherence to medications as a way for patients to defray out-of-pocket expenses [27]. Patients with severe financial distress may also have worse clinical outcomes and increased mortality [28]. Cost of breast cancer chemotherapy varies widely by regimen, and even insured patients have a substantial financial burden [29]. In addition to the direct costs of treatment, indirect costs to patients may include complications and toxicities associated with CT, which may lead to hospitalization, and which also vary by regimen [30]. Although there may be substantial clinical benefit to include CT in treatment plans for patients at high risk of distant recurrence, some patients may feel the benefits do not outweigh the potential complications, long-term adverse effects, and high financial burden.

Future studies may benefit from further investigation into the reasons that 70-GS High Risk patients choose not to include CT as part of their cancer treatment, and to further elucidate long term benefits in various clinical settings. Related, a limitation of this study is that patients were enrolled both prior to and following the publication of the MINDACT trial results, which potentially could have affected physicians’ treatment decisions. The majority of IMPACt patients were enrolled after the publication of the MINDACT study [6], and a comparison of treatment recommendations pre- and post-MINDACT revealed no significant difference in frequency of concordance with 70-GS results, either in the 70-GS Low Risk or High Risk groups. In the 70-GS Low Risk group enrolled after the MINDACT publication, 92.6% of treatment recommendations were concordant with 70-GS results, suggesting a high level of confidence in 70-GS results to safely forego recommending CT for these patients. Although the trial design of this impact study did not include long-term clinical outcome data for these patients, longer follow-up in the MINDACT trial will likely provide additional data on the benefit or lack of benefit of CT in the randomized patient groups.

## Conclusions

The IMPACt trial showed that the majority (88.5%) of treatment plans were concordant with 70-GS results, indicating that physicians make treatment decisions based on the 70-GS result in clinical practice. Physicians also reported an increase in confidence in 72.2% of their recommended treatment plans after receiving the 70-GS results. These results are similar to the reported change in physician confidence in treatment (78.6%) in the PROMIS trial [10]. Taken together, these findings suggest that physicians feel the appropriate patients (High Risk) are being offered chemotherapy, and they feel comfortable sparing 70-GS Low Risk patients from the high clinical and financial burden of chemotherapy [25, 28, 31]. Avoiding overtreatment and the adverse effects of chemotherapy regimens, including hospitalizations [30, 31], lower quality of life, and high financial burden [24, 26, 27, 31], in patients who are unlikely to derive meaningful clinical benefit [6] is of substantial value.

## Supplementary information

**Additional file 1: Table S1.** Molecular subtyping compared with clinical subtyping results.

## Data Availability

All data relevant to this report are included in this published article and its supplementary information files. The datasets analyzed during the current study are not publicly available due to protection of participant privacy and confidentiality but are available in anonymized form from the corresponding author on reasonable request.

## Abbreviations

70-GS: 70-gene signature
80-GS: 80-gene signature
CT: Chemotherapy
HR: High Risk
LR: Low Risk
