# Supplementary material for: MammaPrint guides treatment decisions in breast Cancer: results of the IMPACt trial
Source: BMC Cancer. 2020 Jan 31;20:81. doi: 10.1186/s12885-020-6534-z (PMC6995096; doi:10.1186/s12885-020-6534-z)
Supplement: Supplementary file 1 — Additional file 1: Table S1. Molecular subtyping compared with clinical subtyping results. [file 12885_2020_6534_MOESM1_ESM.docx]

**Additional file**

Soliman et al. **MammaPrint Guides Treatment Decisions in Breast Cancer: results of the IMPACt trial**

**Materials and Methods**

Clinical Subtyping Assessment

Clinical subtyping assessment was performed using a combination of pathological factors, including ER, PR, HER2, and Ki67 status, according to St. Gallen guidelines [1] for the definitions of intrinsic subtypes of breast cancer. ER, PR, HER2, and Ki67 statuses were determined by the local institution according to American Society of Clinical Oncology and College of American Pathologists guidelines for testing [2, 3]. No attempt to achieve harmonization was made among the 18 participating centers. ER and PR were considered positive if ≥1 percent of tumor cells demonstrated positive nuclear staining by IHC, as determined by institutional pathology laboratory assessment. Luminal A-like tumors were defined as: ER+, PR+ (≥20%), HER2-negative and Ki-67-low (<14%). Luminal B-like tumors were defined as ER+, HER2-negative and either Ki67-high (≥14%) or PR-low/negative (<20%). Tumors are also considered Luminal B if HER2 is amplified and ER is positive (regardless of Ki-67 or PR expression). HER2-positive tumors include those in which HER2 is IHC 3+ or FISH/ISH amplified and ER and PR are absent (<1 percent of tumor cells with positive staining). Basal-like/triple-negative tumors should be negative for ER, PR, and HER2 (0-1+ IHC or FISH/ISH non-amplified).

**Supplementary Results**

**Table S1. Molecular subtyping compared with clinical subtyping results.**

|  | **Molecular Subtype (80-GS + 70-GS Results)** | | | |  |
| --- | --- | --- | --- | --- | --- |
| **Clinical Subtype** | **Luminal A-type** | **Luminal B-type** | **ERBB2 (HER2)-type** | **Basal-type** | **Total** |
| Luminal A-type | 158 (72.8%0 | 58 (26.7%) | 0 (0%) | 1 (0.5%) | 217 |
| Luminal B-type | 75 (47.5%) | 67 (42.4%) | 2 (1.3%) | 14 (8.9%) | 158 |
| HER2-type | 0 (0%) | 0 (0%) | 4 (80.0%) | 1 (20.0%) | 5 |
| Triple Negative-type | 1 (25.0%) | 0 (0%) | 0 (0%) | 3 (75.0%) | 4 |
| **Total** | 234 (60.9%) | 125 (32.6%) | 6 (1.6%) | 19 (4.9%) | 384 |

**Molecular subtype classification by 80-GS compared with conventional subtype assessment.**  Results of clinical subtyping of tumors compared to classification by molecular subtyping are summarized in Supplementary Table 1. This analysis included patients regardless of receptor status (hormone receptor-positive or negative, HER2-positive or negative) or treatment setting (adjuvant or neoadjuvant) (n=384 patients). The 80-GS reclassified 14/158 (8.9%) Luminal B tumors as Basal-type and 2/158 (1.3%) Luminal B tumors as HER2-type. One of five tumors classified as HER2-type by clinical pathology was reclassified to Basal-type by the 80-GS, and one tumor that was HER2+ by pathology, which was also ER+ and thus classified to clinical Luminal B, reclassified to Luminal A by molecular subtyping. One of 217 tumors (0.5%) classified as Luminal A by clinical pathology was classified as Basal-type by the 80-GS. One of four (25.0%) tumors classified as triple-negative by clinical pathology was reclassified as Luminal A by molecular subtyping. The total frequency of subtype reclassifications was 39.3% (n=152/384).

**References**

1. Goldhirsch A, Winer EP, Coates AS, Gelber RD, Piccart-Gebhart M, Thurlimann B, Senn HJ, Panel M. Personalizing the treatment of women with early breast cancer: highlights of the St Gallen international expert consensus on the primary therapy of early breast Cancer 2013. Ann Oncol. 2013;24(9):2206–23.

2. Hammond ME, Hayes DF, Dowsett M, Allred DC, Hagerty KL, Badve S, Fitzgibbons PL, Francis G, Goldstein NS, Hayes M, et al. American Society of Clinical Oncology/College of American Pathologists guideline recommendations for immunohistochemical testing of estrogen and progesterone receptors in breast cancer (unabridged version). Arch Pathol Lab Med. 2010;134(7):e48–72.

3. Wolff AC, Hammond ME, Hicks DG, Dowsett M, McShane LM, Allison KH, Allred DC, Bartlett JM, Bilous M, Fitzgibbons P, et al. Recommendations for human epidermal growth factor receptor 2 testing in breast cancer: American Society of Clinical Oncology/College of American Pathologists clinical practice guideline update. J Clin Oncol. 2013;31(31):3997–4013.
